# Comparison of a virtual reality compression-only Cardiopulmonary Resuscitation (CPR) course to the traditional course with content validation of the VR course – A randomized control pilot study

**DOI:** 10.1016/j.amsu.2022.103241

**Published:** 2022-01-05

**Authors:** Dalal Hubail, Ankita Mondal, Ahmed Al Jabir, Bijendra Patel

**Affiliations:** a Bart's Cancer Institute, Queen Mary University of London, UK; b Upper Gastrointestinal Surgeon, Royal London Hospital, UK

**Keywords:** VR, Virtual Reality, CPR, Cardiopulmonary Resuscitation, CVD, Cardiovascular Disease, UK, United Kingdom, Cardiopulmonary resuscitation (CPR), Virtual reality (VR), Skill acquisition, Content validation

## Abstract

**Introduction:**

Technology has been a major contributor to recent changes in education, where simulation plays a huge role by providing a unique safe environment, especially with the recent incorporation of immersive virtual reality (VR) training. Cardiopulmonary Resuscitation (CPR) is said to double, even triple survival from cardiac arrest, and hence it is crucial to ensure optimal acquisition and retention of these skills. In this study, we aim to compare a VR CPR teaching program to current teaching methods with content validation of the VR course.

**Methods:**

A randomized single-blinded simulation-based pilot study where 26 participants underwent baseline assessment of their CPR skills using a validated checklist and Laerdal QCPR®. Participants were randomly allocated and underwent their respective courses. This was followed by a final assessment and a questionnaire for content validation, knowledge and confidence. The data was analysed using STATA 16.2 to determine the standardized mean difference using paired and unpaired *t*-test.

**Results:**

Subjective assessment using the checklist showed statistically significant improvement in the overall scores of both groups (traditional group mean improved from 6.92 to 9.61 *p-value* 0.0005, VR group from 6.61 to 8.53 *p-value* 0.0016). However, no statistically significant difference was noted between the final scores in both the subjective and objective assessments. As for the questionnaire, knowledge and confidence seemed to improve equally. Finally, the content validation showed statistically significant improvement in ease of use (mean score 3 to 4.23 *p*-*value* of 0.0144), while for content, positivity of experience, usefulness and appropriateness participants showed similar satisfaction before and after use.

**Conclusion:**

This pilot study suggests that VR teaching could deliver CPR skills in an attractive manner, with no inferiority in acquisition of these skills compared to traditional methods. To corroborate these findings, we suggest a follow-up study with a larger sample size after adding ventilation and Automated External Defibrillator (AED) skills to the VR course with re-examination after 3–6 months to test retention of the skills.

## 1 Summary

Numerous studies have been conducted over the years to determine new methods in teaching CPR skills, however, the use of virtual reality in this field has been limited, although it's usefulness in education has been proven in many other fields.

This study suggests that the use of immersive virtual reality conveys high quality CPR skills in a attractive and innovative manner.

## 2 Introduction

Cardiac arrests are a huge burden on the global society. The number of out-of-hospital cardiac arrests is highest in North America followed by Europe, Australia and finally Asia, however, Asia has the lowest survival rates [1]. According to the British Heart Foundation (BHF) there are about 7 million people with cardiovascular disease (CVD) in the UK, with one person dying every 3 min [2]. The healthcare cost of CVD is £9 billion, while the cost to the UK economy (in ways such as disability) is £19 billion [2]. It is estimated that 53 per 100,000 population suffer from cardiac arrests in England with 7.9% survival to hospital discharge [3].

In managing cardiac arrest, guidelines have stated the so-called “Chain of Survival” [4], with each link contributing to the ultimate survival of the patient. A crucial step is early CPR, where many studies have demonstrated that high-quality CPR improves survival from cardiac arrest, some studies even quote it as doubling the chances of survival [5].

However, even with all the evidence advocating the importance of CPR, the rate of bystander CPR and the quality of both in and out of the hospital CPR is still low [6,7]. This is mainly attributed to the poor retention of these skills after traditional courses [8,9]. Bhanji et al. conducted a literature review that showed that the suboptimal training was one of the reasons that led to deterioration of these skills as early as 3 months after the course [10]. Furthermore, current CPR training seems to be accessible only to specific sectors of the population [11].

Hence, a lot of research has been done in attempts to find ways to improve acquisition of these skills and to prevent or at least minimize deterioration. Over the years many new methods for teaching CPR have been developed, including self-instruction videos [11,12], peer training schemes [13], compression-only CPR [14] and feedback devices [15]. However, none of these studies found a superior method of teaching CPR, therefore, CPR quality has remained suboptimal [[11], [12], [13], [14], [15]]. Furthermore, studies looked into enhancing retention of these skills, such as self-retraining [16] or student directed retraining [17]. However, none of these have shown drastic improvements beyond traditional teaching methods.

Therefore, we aim to look at a new method of delivering these crucial skills through technology-enhanced learning, as it is currently an important tool in healthcare education [18]. We specifically look at Virtual Reality training (VR), which has been named, ‘the teaching tool of the twenty first century’ [19]. It is being incorporated into many different disciplines, including language, philosophy, architecture, special education and medical education. In healthcare education specifically, VR training has been studied at length in different surgical specialities and one common conclusion was reached, i.e. VR improves skill acquisition and decreases intraoperative time [[20], [21], [22], [23], [24], [25]]. A review of literature by Kuyt et al. about the use of VR and augmented reality in CPR training concluded that these have great potential, however, more studies are crucial to consolidate the findings and encourage introduction of new innovations into healthcare education [26].

Furthermore, when assessing CPR skills it is crucial that any new method of teaching doesn't risk swaying away from evidence based guidelines. Virtual reality has been shown to sustain adherence to protocols, even improve them, according to the American Heart Association guidelines [27].

CPR is a crucial skill, for medical and non-medical personnel, which could save lives and ease our jobs as healthcare providers. Introducing technology such as VR could ease the delivery of the skill and attract more people to learn and use this crucial skill. Hence, the purpose of this pilot study is to compare compression only CPR skill acquisition in the VR course versus the traditional course, with content validation of the VR CPR course.

## 3 Methods

This is a randomized simulation-based pilot study, with two groups of participants: the traditional teaching group being the control group, while the VR teaching group is the study group. This trail is registered at *The Research Registry*, number *researchregistry7262*.

No ethical approval was needed, as this study is a simulation-based study on healthy volunteers with no access to confidential information.

### 3.1 Recruitment of Participants

As this is a pilot study, power calculations were not necessary at this stage. Participants were recruited based on the following inclusion and exclusion criteria.

Inclusion Criteria:

1. Adult ≥ 18 years old
2. Never did a CPR course before *OR* if previously done, was more than a year ago

Exclusion Criteria:

1. Recent (<1 year) CPR training, including Basic or Advanced Life Support
2. Unable to perform physical skills required for any CPR course such as being pregnant or having certain back/joint problems

### 3.2 Participants recruited

A total of 42 individuals met the inclusion criteria and therefore were contacted. 35 showed willingness to participate and 7 volunteers refused participation in the trial due to other prior commitments. On the day, 26 participants attended and 7 dropped out for various reasons, including illness.

### 3.3 Method of randomisation

**Fig. 1:**
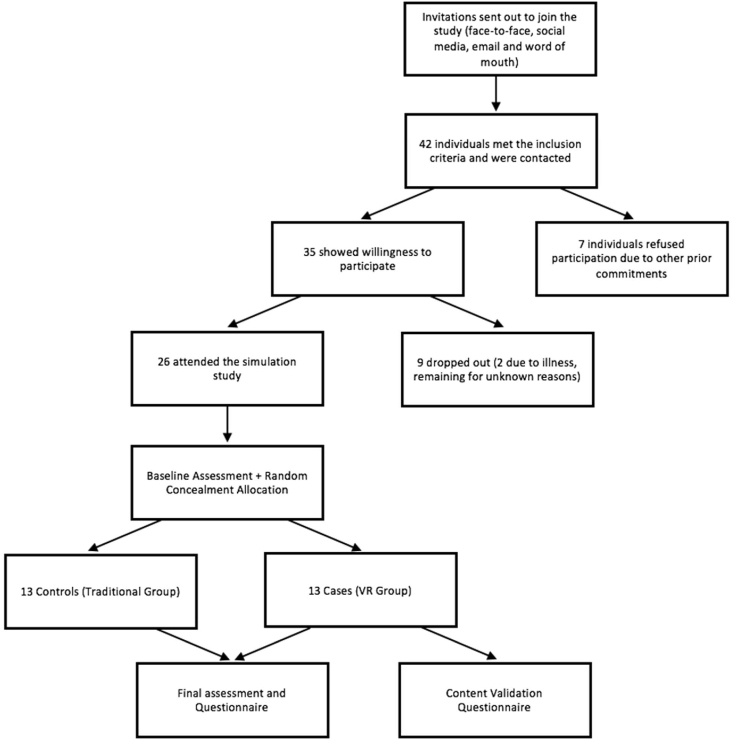
CONSORT diagram showing Recruitment of Participants.

Random allocation with concealment (envelope) was used to randomly allocate an equal number of participants in each group, 13 in the traditional group and 13 in the VR group (shown in Fig. 1). This allocation was known to the principle investigator and statistician, but the examiner was blinded.

**Fig. 2:**
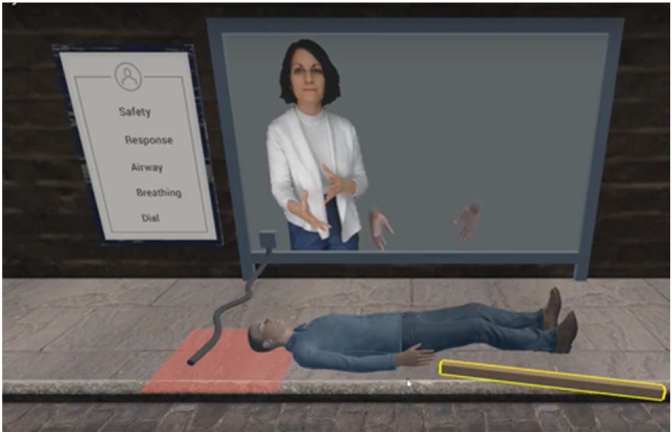
Initial screen of VR course.

### 3.4 Data collection

Every participant underwent a baseline objective and subjective assessment of their CPR skills using Laerdal QCPR® feedback manikin (compression rate, depth and accuracy of chest recoil) and an assessment checklist (adopted from Resuscitation Council UK, shown in Table 1) respectively. This lasted around 3–5 min, with no direct feedback to the participants from the examiner or the manikin. Then they proceeded to their respective courses.

1. *Traditional Course:* This is a certified instructor led course that is divided into 2 sections: lectures and hands-on skills. The lecture details the steps of CPR and what is expected with different scenarios. Furthermore, the hands-on section entails applying the information from the previous section on a CPR feedback manikin. This course has one instructor with a group of 12–15 participants usually lasting around 4 h with an assessment at the end. Each 3–4 participants shared a manikin to practice within a limited time, as these courses usually entail other topics, such as choking.
2. *VR course:* This course was developed by a company called *dualgoodhealth.* It is led by an experienced instructor, where the participant wears a VR headset and hand sensors (HTC Vive) then follows verbal and visual instructions focusing more on the hands-on experience on a CPR manikin. The software marks the steps done (as shown in Fig. 2 showing the initial VR screen) and gives both verbal and visual feedback if performance is suboptimal. A single session takes around 5–7 min and was conducted twice for each participant.

**Table 1:** Criteria used in the Assessment Checklist.

1. Ensures safety of patient and rescuer 2. Checks for responsiveness (taps and talks to patient) 3. Opens airway: performs head tilt and chin lift 4. Assesses breathing: no breathing or only gasping 5. Assesses pulse: no pulse felt within 5–10 s (done simultaneously with assessment of breathing) 6. Shouts out for help and an Automated External Defibrillator (AED) 7. Calls the emergency services 8. Chest Compressions: • Correct hand placement (2 hands on the lower half of the sternum) • Rate of 100–120 per minute • Depth of 5–6 cm • Allows full chest recoil after each compression • Minimizes interruptions (to less than 10 s) 9. Correct sequence of actions

After completing their respective courses each participant underwent a subjective and objective final assessment identical to the baseline assessment using the same checklist and Laerdal QCPR® feedback manikin by the same blinded examiner. Finally, all participants answered a questionnaire regarding methods of learning/teaching, concerns and confidence performing CPR, in addition to basic CPR theory (Details in Table 2), while only the VR group answered the content validation questionnaire (Details in Table 3) using a 5-point Likert Scale.

**Table 2:** Background, confidence/concerns and theory questionnaire (using 5-point Likert scale).

1. Demographics: Name, Age, Gender, Level of Education, Occupation, Days since last CPR training if any 2. Have you ever used Virtual Reality (VR) headset in any setting (including video games)? If yes, in what context and how was your experience with it? 3. I think that the VR headset and Technology Enhanced Learning in education and training is: widely used, occasionally used, not used, not available, not well developed 4. Preferred method of teaching: Textbooks, Lectures, Audio-visual, Live demonstrations, Hands-on training or Technology-enhanced learning (Virtual Reality) 5. Do you feel confident to perform CPR after taking this course? 6. Concerns regarding CPR • I fear of further harming the victim by performing CPR • I would more likely initiate CPR if it was a young victim • The victim being of the same gender would affect me • The victim being of the opposite gender would affect me • The victims being from different ethnicity or country would affect me • The fear of contracting a disease from the victim affects me • Knowing the victim personally would positively affect my decision to start CPR • The worry about legal liability affects my decision to perform CPR 7. CPR Theory • In which of the following scenarios would you stop CPR? • What is the correct rate of chest compressions? • What is the correct depth of chest compression? • What is the correct hand position during CPR? • What would you do when the patient regains his pulse?

**Table 3:** VR content validation questionnaire (using 5-point Likert scale).

1. Before using the VR software • Ease of Use • Realistic Feel of the Virtual Environment • Usefulness of VR • Appropriateness of VR • Positive and fun experience 2. After using the VR Software • Ease of Use ○ VR felt natural with no extra effort ○ I was immersed in the virtual environment that I was not aware of what was going on around me ○ The equipment used (headset/hand piece) did not interfere with the task ○ Fairly straightforward ○ Overall easy to use • Content ○ The instructions are clear and understandable ○ The feedback is clear and useful ○ Adjustments to performance were made based on live feedback ○ Professional appearance/design of the VR environment ○ The virtual environment felt realistic • Usefulness of VR • Appropriateness of VR • Positive and fun experience • If freely available, I would use VR to enhance my learning experience • I feel confident to perform BLS in a real-life situation after taking this course

### 3.5 Data analysis

The collected data was analysed using STATA version 16.2 for both the subjective and objective assessments, where a paired and unpaired *t*-test was conducted to compare the standardized mean differences with a significance level of α < 0.05. As for the questionnaire, a comparison was done between the two groups using an unpaired *t*-test to assess the confidence to perform CPR and CPR knowledge gained after each training session (*p-value* of <0.05 was considered statistically significant). Finally, the content validation questionnaire was analysed using standardized mean difference with *p-value* generated using the paired *t*-test (statistically significant *p-value* < 0.05).

## 4 Results

The participant pool was majority female (traditional = 61.5%, VR = 84.6%) with a mean age of 24.85 years (SD 3.85) in the traditional group and 23.15 years (SD 5.19) in the VR group. As per the inclusion and exclusion criteria, participants should have no previous CPR training or their last training was more than a year ago; more than half the participants in each group had no previous training (traditional = 53.8%, VR = 69.2%). The remaining participants in each group that did have previous training (traditional = 46.2%, VR = 30.8%) had a mean of 2.58 years since last training in the traditional group while it was 3.25 years in the VR group.

The comparison of baseline skills demonstrates no statistically significant differences between the two groups using both the subjective or objective assessments verifying both groups started at similar skill levels.

### 4.1 Subjective assessment

A comparison between the baseline and final assessment checklists in both groups was conducted using paired *t*-test and showed statistically significant (*p-value* of <0.05) change in mean score. In the traditional group, the mean score increased from 6.92 to 9.61, while in the VR group it increased from 6.61 to 8.53 (Table 4). Furthermore, the standardized mean difference (SMD) was 2.69 in traditional group, while it was 1.92 in the VR group, which are statistically significant differences. However, a comparison between the final scores of both groups using unpaired *t*-test showed no statistically significant difference in the scores (*p-value* 0.089), which implies that the CPR skill acquisition may be comparable in both groups with a standardized mean difference of −1.07, which is statistically not significant.

**Table 4:** Comparison of checklist baseline and final overall scores.

	Baseline Score		Final Score		SMD	*p*-*value*
	Mean	SD^a^	Mean	SD^a^		
Traditional Group	6.92	2.87	9.61	1.66	2.69	**0.0005**
VR Group	6.61	3.06	8.53	2.25	1.92	**0.0016**

a SD: Standard Deviation.

### 4.2 Objective assessment

A comparison of the final results in all three variables measured in the QCPR was conducted between the two groups using an unpaired *t*-test, which showed no statistically significant (*p*-value of ≥ 0.05) difference (Table 5). The chest compression rate (SMD -2.61), depth of compressions (SMD -2.15) and chest recoil (SMD 5.23) showed no difference between the two groups. This further emphasizes the fact that the VR and Traditional teaching methods could have similar impacts on CPR training.

**Table 5:** Comparison of Laerdal QCPR mean scores between the final scores of the two groups.

	Traditional	VR	Combined Mean	SMD^a^	*p-value*
Compression Rate (/min)	113.69	111.07	112.38	−2.61	0.3586
Depth of Compression (mm)	47.23	45.07	46.15	−2.15	0.2132
Chest Recoil (%)	78.15	83.38	80.76	5.23	0.3291

a SMD: Standardized Mean Difference

### 4.3 Knowledge and confidence/concerns questionnaire

All participants were asked about general learning preferences and use of technology enhanced learning. Around 65.4% of the participants have never used a VR headset before, with the remaining 38.5% having used it in gaming. As for use of VR and technology enhanced learning in current teaching 42.3% thought it was occasionally used, while 23.1% chose not used and not available. Furthermore, participants indicated that their preferred method of teaching/learning is hands-on training with a mean scoring of 4.62 on a 5-point Likert Scale. Followed by live demonstrations (mean = 4.27) and Immersive Learning/VR (mean = 3.85), with textbooks ranking last with a mean of 2.50.

To further evaluate the knowledge acquired during these courses, we asked the participants to answer a few multiple choice questions regarding basic CPR facts. There was only a slight difference in the mean scores between the groups (traditional = 4.69, VR = 4.00) that is not statistically significant (*p-value* 0.083).

Finally, all participants were asked if they felt confident performing CPR after taking these courses: The traditional BLS group had a mean of 3.76 on a 5-point Likert scale while the VR had a mean of 3.46. There was no statistically significant difference between the two groups (*p-value* 0.782). As a follow up to this, we addressed some of the possible concerns in regards to performing CPR. Most people were not affected by the fear of contracting a disease, gender or ethnicity of the victim. However, a few were worried about legal liability and further harming the victim with a mean of 2.04. Finally, most people agreed that knowing the victim would encourage them to start CPR quicker with a mean score of 3.12.

### 4.4 Content validation

A comparison was done between the views of the participants in regard to VR before and after the course using a paired *t*-test. The only statistically significant difference in opinion before and after VR use was in overall ease of use. For overall ease of use the mean went from 3 before to 4.23 after (*p*-*value* 0.0144, which is statistically significant). As for content, usefulness, appropriateness and positivity of the experience, scores were similar before and after use (Table 6). Most participants found that it was a positive and fun experience with a mean of 4.07, however, usefulness was ranked less at 3.61. This maybe due to the fact that the VR scored lower on the naturality of the environment (mean score = 2.46) with some interference from the equipment used (mean score = 3.31). Nevertheless, overall content scored very high with a mean of 4.15, with its main subdivisions scoring the following: clarity of instructions (mean score = 4.46), usefulness of the feedback (mean score = 4.31) and appearance of the VR environment (mean score = 4.31). Finally, participants after the VR experience gave a mean score of 3.92 for using VR to enhance their learning in the future if available.

**Table 6:** Content validation of the VR course.

	Mean (Before)^a^	SD	Mean (After)^a^	SD	SMD	*p*-value
**Overall ease of use**	**3.00**	**1.22**	**4.23**	**0.83**	**1.23**	**0.0144**
Overall content	3.38	1.26	4.15	0.69	0.76	0.0626
Usefulness of VR	3.46	1.05	3.61	1.38	0.15	0.4039
Appropriateness of VR	3.53	0.96	3.69	1.10	0.15	0.3798
Positive and fun experience	4.46	0.51	4.07	1.11	−0.38	0.1338

a Before and After taking the VR CPR course.

## 5 Discussion

A vast amount of research has been conducted over the years looking at methods to improve widespread acquisition of CPR skills, as currently 77% of people in the UK are either not trained or not confident in performing CPR [28]. It has been shown that CPR doubles maybe even triples chances of survival post-cardiac arrest [28]. This extends from looking into methods of teaching the elderly population, since most cardiac arrests do occur at home [3] to teaching high school, or even younger, students [29]. Furthermore, research has been conducted looking at the different methods of delivering these skills and trying to find ways to make it easier, quicker and more efficient. These include self-teaching videos [11], ‘hands-only’ CPR [30], and real-time training software [31]. However, we are still lagging behind and better more efficient methods of training are needed, and hence we aimed at presenting an efficient and memorable way of teaching these crucial skills using immersive virtual reality.

The idea of using VR for CPR training has been discussed for the past few years. Initially, in 2009 a study conducted by Semeraro et al. [32] was published addressing the ‘acceptance’ of resuscitation experts of VR enhanced manikins. Later on, a follow up study was done as a 5-question survey to instructors around the globe with responses from 18 countries about current attitudes about VR in CPR training and its future prospects. This survey showed that 72.8% of instructors believed VR will be part of the future of CPR training [33]. Furthermore, a review of available literature was done which showed that VR and augmented reality have great potential in CPR training, but more studies are needed to fully establish it's role [26]. Hence, our results add to this available literature by showing no inferiority in the aquisition of CPR skills between VR courses and the traditional teaching methods.

As for the questionnaire, it was designed by amalgamating preexisting validated questionnaires from literature [34,35]. Participants were initially asked about their preferred teaching/learning style where hands-on got the highest ranking followed by live demonstrations and technology enhanced learning, which are all components incorporated within the immersive virtual reality CPR course. Then participants were asked about concerns and confidence performing CPR, where the only significant finding was a positive correlation between knowing the person and starting CPR. This could be incorporated into the VR learning scheme in the future as personalized avatars to make the environment more realistic. As for the confidence, both groups had a similar degree of conferred confidence after the course. This is crucial as only 22% of the 47% of the UK population trained in CPR feel confident performing it [28]. Immersive virtual reality has been shown in literature to be a great way to boost confidence, motivation and encourage people to apply taught skills to real-life situations [36]. Finally, the last part of the questionnaire involved the content validation of the VR course. Overall, the mean scores for all categories were high, with a statistically significant change in ease of use indicating that it’s much simpler to use than people imagine. This emphasizes that this software is a great start to what could be a game changer in CPR training.

Based on the above findings, VR provides us with a possible adjunct to the traditional teaching with a few advantages. These include the appeal the VR teaching method has on the younger generation, where it has been shown that young individuals are more likely to retain CPR skills and share this knowledge with people around them [29].

Furthermore, it is important to emphasise that with VR the sessions require a short period of time (5–7 min), which may encourage people to take time out of their busy days to learn these essential skills and make it feasible to give each person more time to practice. Repetitive testing has shown a trend of slower decay of skills [37], so we suggest the option of giving temporary online access to the software after 6 months to use as a refresher with low cost material, such as google cardboard VR glasses with cardboard manikins. The use of such low-cost material was attempted in a study by Wik et al. [13] looking at a peer teaching scheme for CPR training, which was found to be cost-effective. An alternative suggestion noticed in literature is the use of a pillow in place of a manikin for the sake of retraining [33]. Moreover, this could address one of the issues with traditional teaching, which is distance and time needed to access such courses, where VR is less time consuming and could be amended to enable remote access. This was shown in the study by Khanal et al. [38] on VR Advanced Cardiac Life Support (ACLS) procedure training which emphasised on the easy accessibility to training when using VR and its influence on the number of participants and retraining opportunities. Furthermore, a study into using a VR application in school children by Yeung et al. has shown promising results in skill acquisition in addition to corroborating the time flexibility and accessibility of such advances in CPR training [39]. This has attracted the attention of many where smartphone applications are being modified to include VR CPR training in the hopes of increasing the number of people with CPR training, such as the application being used in the *Lowlands Saves Lives trial* [40]. This is even more crucial at present with the COVID-19 pandemic and its impact on accessibility to training [41].

In addition, monitoring of CPR quality, including feedback simulation training, is recommended because it increases likelihood of high-quality CPR, which translates into improved outcomes [42]. Based on the review done by Yeung et al. [15] concluding that use of feedback systems improves skill acquisition and retention came the recommendation by the international Liaison Committee on Resuscitation to develop new feedback systems [43]. Furthermore, a study by Couper et al. [44] suggested that real-time feedback may help slow down the deterioration of skills. Therefore, this further validates the important aspect of the VR technology which gives live visual and verbal feedback throughout the training to ensure maximum acquisition and retention of skills.

Focusing specifically on our results and the VR CPR course available, our main limitation was the lack of certain aspects from the software, namely ventilation and Automated External Defibrillator (AED) AED skills. Hence, the VR CPR course can be improved by adding both breathing and AED as a start to move away from compression only CPR to CPR with the 30:2 compression:ventilation ratio, which is the recommended current best practice [30]. The current teaching scheme remains 30:2 (chest compressions:ventilation) due to lack of strong evidence to change to compression-only, however, the debate continues on whether bystander CPR would be better taught as compression-only with some studies showing that people are more likely to start compression-only CPR rather than 30:2 [45]. Hence, there is a current recommendation by the International Consensus on CPR and Emergency Cardiovascular Care Science with Treatment Recommendations (ILCOR CoSTR) that dispatchers at least encourage bystanders – who may not be trained or willing to do 30:2 – to start compression-only CPR, until help arrives [43]. Also, the software only addresses adult CPR and one-rescuer CPR whereas traditional CPR courses address child/infant CPR which has its unique rules, such as position of the hands in infants, in addition to two-rescuer CPR [4].

Another limitation is the small sample size, however, as it is a pilot study it did not necessitate power calculations. Nevertheless, the number we had gave us enough information to come to initial conclusions regarding the VR training course and its importance as a training tool, in addition to giving us a baseline for future power calculations.

Overall, both VR and the traditional teaching methods showed similar teaching capabilities with improvement in skills noted in the final scores of the assessment checklist of both groups with comparable final results in both the objective and subjective assessments. This implies that both methods may deliver CPR skills effectively. However, a larger scale follow up study with the addition of the missing components and long term follow up is needed to establish the scale of skill acquisition and retention.

## 6 Conclusion

1. The VR teaching method is an appealing approach to teaching in general and is useful for introducing new topics such as CPR to the general public. This pilot study suggests that VR teaching could deliver CPR skills in an attractive manner, with no inferiority in acquisition of these skills compared to traditional methods.
2. In the future, it would be very useful to corroborate these findings by performing a follow-up study with a larger sample size after the addition of ventilation and AED training components with re-examination after 3–6 months to assess the long term impact VR training has on CPR skill acquisition and retention.
3. Finally, immersive virtual reality is definitely where teaching is heading in the twenty first century, and it has shown great promise in maximising learning and motivation. Hence, for CPR, after the addition of the components mentioned previously, virtual reality could be a great adjunct to CPR training.

## Provenance and peer review

Not commissioned, externally peer reviewed.

## Ethical approval

No ethical approval needed as simulation study.

## Sources of funding

None.

## Author contribution

Dalal Hubail - Primary Author, Design and Implementation of Research (including running the courses, analysis), writing the manuscript.

Ankita Mondal - Contributed to running the courses and design.

Ahmed Al Jabir - Contributed to running the courses and data analysis.

Prof Patel - Supervisor and advisor.

## Research registration Unique Identifying number (UIN)

1. Name of the registry: The Research Registry
2. Unique Identifying number or registration ID: researchregistry7262
3. Hyperlink to your specific registration (must be publicly accessible and will be checked): https://www.researchregistry.com/browse-the-registry#home/

## Guarantor

Dalal Hubail.

Professor Bajendra Patel.

## Declaration of competing interest

None.
